# Cutaneous Manifestations as a Sentinel of Colorectal Cancer: A Case Report

**DOI:** 10.3390/jcm15072789

**Published:** 2026-04-07

**Authors:** Bárbara Marinho, Glória Velho, Marisa D. Santos

**Affiliations:** 1 Digestive and Extradigestive Service, Surgery Department, Centro Hospitalar Universitário de Santo António, Unidade Local de Saúde de Santo António, 4099-001 Porto, Portugal; u12383@ulssa.min-saude.pt; 2 Dermatology Service, Centro Hospitalar Universitário de Santo António, Unidade Local de Saúde de Santo António, 4099-001 Porto, Portugal; gloriacunhavelho@gmail.com; 3 Colorectal Surgery Unit, Digestive and Extradigestive Surgery Service, Centro Hospitalar Universitário de Santo António, Unidade Local de Saúde de Santo António, 4099-001 Porto, Portugal; 4 ICBAS—School of Medicine and Biomedical Sciences, University of Porto, 4050-313 Porto, Portugal; 5 UMIB (Unit for Multidisciplinary Research in Biomedicine), ICBAS—School of Medicine and Biomedical Sciences, University of Porto, 4045-313 Porto, Portugal; 6 ITR (Laboratory for Integrative and Translational Research in Population Health), 4050-600 Porto, Portugal

**Keywords:** erythema gyratum repens, paraneoplastic syndrome, paraneoplastic dermatosis, colorectal cancer, colon adenocarcinoma, cutaneous manifestations, cutaneous marker of malignancy, case report

## Abstract

Erythema gyratum repens (EGR) is a rare figurate erythema strongly associated with internal malignancy and recognized as one of the most specific cutaneous paraneoplastic syndromes. Its recognition is clinically important, as it frequently precedes the diagnosis of an underlying neoplasm. We report the case of an 80-year-old woman who presented to the emergency department with a rapidly progressive, intensely pruritic eruption displaying a characteristic concentric “wood-grain” pattern. Laboratory evaluation revealed iron-deficiency anemia. Contrast-enhanced computed tomography identified a right-sided colonic mass, and colonoscopy with biopsy confirmed adenocarcinoma of the cecum. The patient underwent elective laparoscopic right hemicolectomy with complete tumor resection (pT3N0, microsatellite stable). Following surgery, the cutaneous lesions resolved completely and did not recur during follow-up. This case highlights erythema gyratum repens as a clinically relevant early marker of colorectal cancer and emphasizes the importance of prompt recognition of this distinctive dermatosis to trigger urgent and comprehensive malignancy screening, enabling timely diagnosis and definitive treatment.

## 1. Introduction

Paraneoplastic syndromes (PNS) are a heterogeneous group of clinical manifestations associated with malignant disease that cannot be explained by direct tumor invasion, metastatic spread, or treatment-related toxicity. Instead, they arise from systemic effects mediated by tumor-derived humoral factors or immune-mediated mechanisms, including cross-reactivity between tumor antigens and normal tissues [1,2]. Importantly, PNS may precede, coincide with, or follow the diagnosis of cancer, occasionally representing the earliest clue to an otherwise occult malignancy [1].

The reported incidence of PNS ranges from approximately 5% to 15% of patients with cancer, although this figure is likely underestimated due to under-recognition and heterogeneous diagnostic criteria [1,2]. Virtually any organ system may be affected, including the endocrine, neurological, hematological, rheumatological, and dermatological systems. Certain malignancies—particularly lung cancer, hematological neoplasms, and gastrointestinal tumors—are more frequently associated with specific paraneoplastic phenomena [2,3].

Clinically, PNSs are highly relevant because they may prompt cancer investigation in patients without an established diagnosis, sometimes preceding tumor detection by months or years [1,3]. In addition, the course of some paraneoplastic syndromes may parallel tumor activity, with improvement after effective oncological treatment and recurrence upon disease relapse, supporting their role as surrogate markers of disease activity [3].

The skin is one of the most commonly involved organs in paraneoplastic syndromes and offers a unique diagnostic advantage, as cutaneous manifestations are readily visible and accessible to clinical examination and biopsy [4]. Several well-characterized paraneoplastic dermatoses have been described, including acanthosis nigricans, dermatomyositis, necrolytic migratory erythema, the Leser–Trélat sign, and figurate erythemas, many of which are associated with gastrointestinal, pulmonary, breast, gynecological, or hematological malignancies [4,5].

Among these entities, erythema gyratum repens (EGR) is a rare but highly distinctive paraneoplastic dermatosis. It is characterized by rapidly migrating, concentric erythematous bands forming a “wood-grain” pattern, typically accompanied by intense pruritus [6,7,8]. Lesions may expand at a rate of up to 1 cm per day and most commonly involve the trunk and proximal extremities, usually sparing the face, hands, and feet [7,8]. Since its original description by Gammel in 1952, EGR has been recognized as one of the cutaneous eruptions most strongly associated with internal malignancy [9].

An underlying neoplasm is identified in the vast majority of reported EGR cases, most frequently lung cancer, followed by esophageal, breast, and gastrointestinal malignancies [6,7,8]. Although less common, an association with colorectal cancer has been consistently reported, with EGR often preceding or coinciding with tumor diagnosis, underscoring its potential value as an early diagnostic marker [10,11,12].

The pathogenesis of EGR remains incompletely understood. Current evidence supports a predominantly immune-mediated mechanism, possibly involving cross-reactivity between tumor antigens and cutaneous structures, immune complex deposition, and cytokine-driven inflammatory responses [5,7]. Histopathological findings are typically non-specific, reinforcing the concept that EGR represents an indirect manifestation of systemic malignancy rather than direct tumor involvement [6,7].

Clinically, EGR frequently improves or resolves following successful treatment of the underlying malignancy, whereas recurrence of the rash has been described in parallel with tumor relapse or progression [6,8,11]. Consequently, EGR may function as a dynamic indicator of tumor activity, rather than an independent prognostic factor.

Considering its rarity but high predictive value for malignancy, recognizing EGR should lead to a systematic search for an underlying cancer, including gastrointestinal assessment when clinically appropriate [6,10].

Although erythema gyratum repens is a well-recognized paraneoplastic dermatosis, this report contributes clinically relevant insights by describing its presentation as the sentinel manifestation of a right-sided, microsatellite-stable colon adenocarcinoma diagnosed in the emergency setting. The case underscores the diagnostic value of dermatologic pattern recognition, highlights the importance of early multidisciplinary assessment, and reinforces the role of EGR as a dynamic marker of tumor activity rather than a purely histopathological entity. This case illustrates how prompt recognition of a distinctive cutaneous pattern can directly influence diagnostic pathways, leading to early detection and curative treatment of an otherwise occult malignancy.

## 2. Case Presentation

In 2023, an 80-year-old woman who was autonomous in daily activities and cognitively intact presented to the Emergency Department of the Centro Hospitalar Universitário de Santo António with a three-week history of a rapidly progressive, intensely pruritic skin eruption. One week earlier, she had been evaluated at another medical facility and treated with oral prednisolone (20 mg/day), hydroxyzine (25 mg/day), and topical betamethasone applied twice daily, without significant clinical improvement.

**Figure 1:**
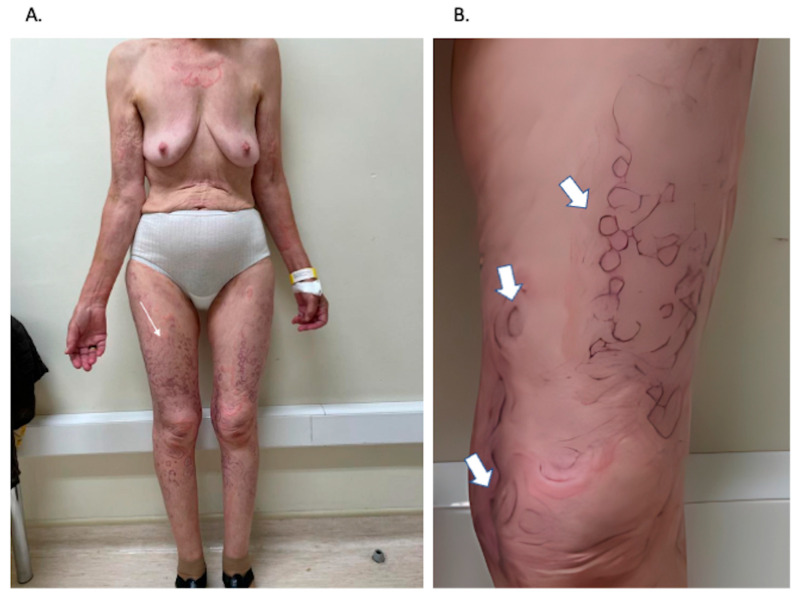
(**A**) Concentric, serpiginous, and annular erythematous–violaceous plaques and macules distributed over the trunk and upper and lower limbs, forming the characteristic “wood-grain” pattern typical of erythema gyratum repens. (**B**) Close-up view of a lesion on the left thigh, highlighting the undulating concentric borders and trailing scale. (Image details: Thin arrow—“wood-grain” pattern; Thick arrows—trailing scale, concentric bands, centrifugal migration).

On admission, dermatological examination revealed annular, serpiginous, and circinate erythematous–violaceous macules and plaques, predominantly affecting the lower limbs and extending to the upper limbs and trunk, forming a characteristic concentric “wood-grain” pattern (Figure 1). Physical examination additionally identified a palpable mass in the right iliac fossa, measuring approximately 5 cm in diameter, with firm consistency and lateral mobility. The patient reported progressive anorexia, general malaise, and unintentional weight loss over several months but denied focal gastrointestinal symptoms, including abdominal pain, overt gastrointestinal bleeding, or changes in bowel habits.

Her past medical history included benign breast nodules under surveillance, chronic gastritis, cystic endometrial hyperplasia, osteoporosis, chronic venous insufficiency, dyslipidemia, and sleep disturbances. She denied any recent infections, vaccinations within the previous 6 months, or exposure to new medications prior to the onset of the rash. A detailed medication review revealed no recent initiation of drugs previously associated with figurate erythemas. There was no history of autoimmune disease or malignancy, and no known drug allergies. Family history was unremarkable for malignancy or autoimmune disorders. Imaging studies performed three months earlier had shown no relevant abnormalities.

Laboratory evaluation revealed iron-deficiency anemia, with a hemoglobin level of 8.9 g/dL and serum iron of 15 µg/dL, without other significant abnormalities. The peripheral eosinophil count was within the normal range (0.24 × 10^3^/µL).

Based on the highly characteristic clinical morphology, a diagnosis of erythema gyratum repens (EGR) was suspected, and the patient was admitted to the Dermatology Department for further investigation. Dermoscopic examination revealed non-specific inflammatory features consistent with active figurate erythema, including a diffuse erythematous background with fine, irregularly distributed linear and dotted vessels. Peripheral whitish scaling was observed at the advancing margins, corresponding to the clinically visible trailing scale. Lesion borders were ill-defined and blurred, consistent with the rapid centrifugal progression typical of EGR. No pigment network, crystalline structures, or features suggestive of infectious or melanocytic dermatoses were identified (Table 1). Fungal studies were not performed, given the highly characteristic clinical presentation and the absence of epidemiological or dermoscopic features suggestive of dermatophytosis.

A skin biopsy was obtained from an active erythematous band on the right arm. Histopathological examination demonstrated a non-specific perivascular dermatitis pattern, which, although not diagnostic, was consistent with previously reported findings in EGR. Direct immunofluorescence was negative for IgA, IgG, IgM, C3, C4c, and fibrinogen. Autoimmune screening, including antinuclear and antineutrophil cytoplasmic antibodies, was negative (see Supplementary Figure S1). Serum protein electrophoresis revealed no relevant abnormalities. Partial symptomatic improvement in pruritus and skin lesions was observed with topical corticosteroids and oral antihistamines; however, persistent disease was anticipated in the absence of treatment for the underlying cause.

**Figure 2:**
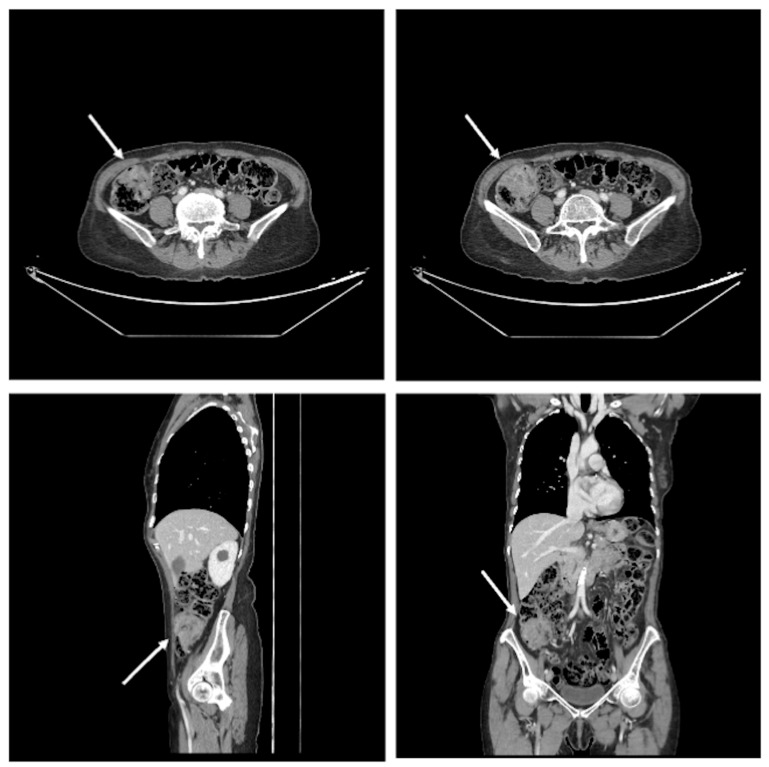
Contrast-enhanced computed tomography scan showing heterogeneous circumferential thickening of the cecum (approximately 5 cm in length), suspicious for malignancy, with small adjacent pericolic lymph nodes and no evidence of distant metastatic disease (arrows pointing to the cecal tumor).

Given the strong paraneoplastic association of EGR and the presence of a palpable abdominal mass, a contrast-enhanced thoracoabdominal–pelvic computed tomography scan was performed. This revealed heterogeneous circumferential thickening of a short segment of the cecum, suspicious for malignancy, with small adjacent pericolic lymph nodes and no evidence of distant metastases (Figure 2).

**Figure 3:**
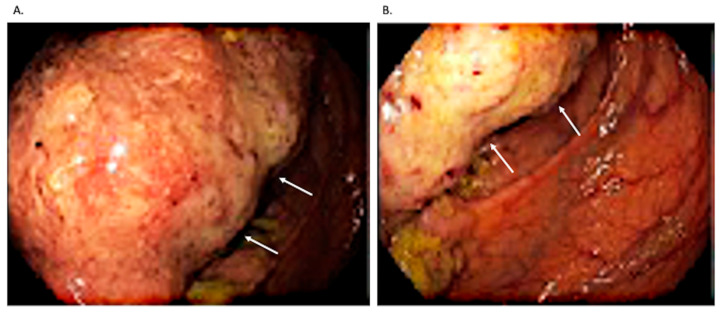
Colonoscopy showing a neoplastic lesion of the cecum involving approximately 75% of the luminal circumference, with ulcerated, sessile, and flat components. Arrows indicate the vegetative component of the tumor. (**A**) Intraluminal tumor mass. (**B**) Tumor implantation base occupies more than 50% of the colonic lumen circumference.

Subsequent colonoscopy demonstrated a neoplastic lesion involving approximately three-quarters of the luminal circumference of the cecum and proximal ascending colon, with ulcerated, sessile, and flat components (Figure 3). Histological analysis of biopsy specimens confirmed colonic adenocarcinoma, while concomitant polyps exhibited adenomatous histology with low-grade dysplasia. Tumor markers were within normal limits (CEA 4.0 µg/L, CA 19-9 16.7 U/mL, CA 72-4 1.7 U/mL).

**Figure 4:**
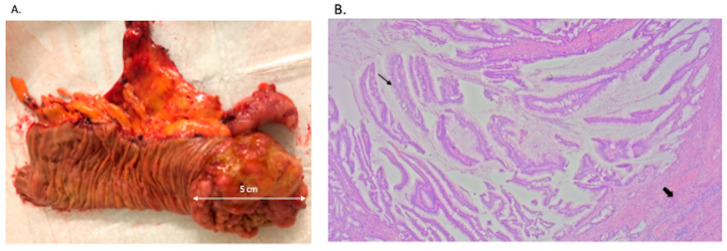
(**A**). Surgical specimen obtained after laparoscopic right hemicolectomy, demonstrating an adenocarcinoma located in the cecum; (**B**). “Adenoma-like” adenocarcinoma. The surface shows a villous architecture with low-grade nuclear atypia (thin arrow). Deep within the wall, there is an invasion of the muscularis propria (thick arrow) (H&E, 25×).

The patient was referred to the Colorectal Surgery Department and maintained on hydroxyzine (25 mg/day) and topical betamethasone twice daily for symptomatic relief. One month later, she underwent an elective laparoscopic right hemicolectomy with intracorporeal anastomosis, without perioperative complications (Figure 4). She was discharged from the Colorectal Unit on postoperative day five.

Histopathological examination of the surgical specimen revealed an adenoma-like adenocarcinoma of the cecum, staged as pT3N0R0 (tumor measuring 5 × 6.5 cm, with 0 out of 22 lymph nodes examined being positive). No invasion of adjacent organs or structures was observed. Lymphovascular invasion was absent, including small vessel involvement as well as intramural and extramural large-vessel (venous) invasion. Perineural invasion was not detected. Tumor budding, evaluated in an area of 0.785 mm^2^, was classified as low-grade (0–4 buds). No tumor deposits, defined as discontinuous extramural tumor extension, were present. This assessment was made according to the AJCC 8th edition, with microsatellite stability. After discussion by the multidisciplinary tumor board, and considering the patient’s age and histologic findings, an active surveillance strategy was implemented.

**Figure 5:**
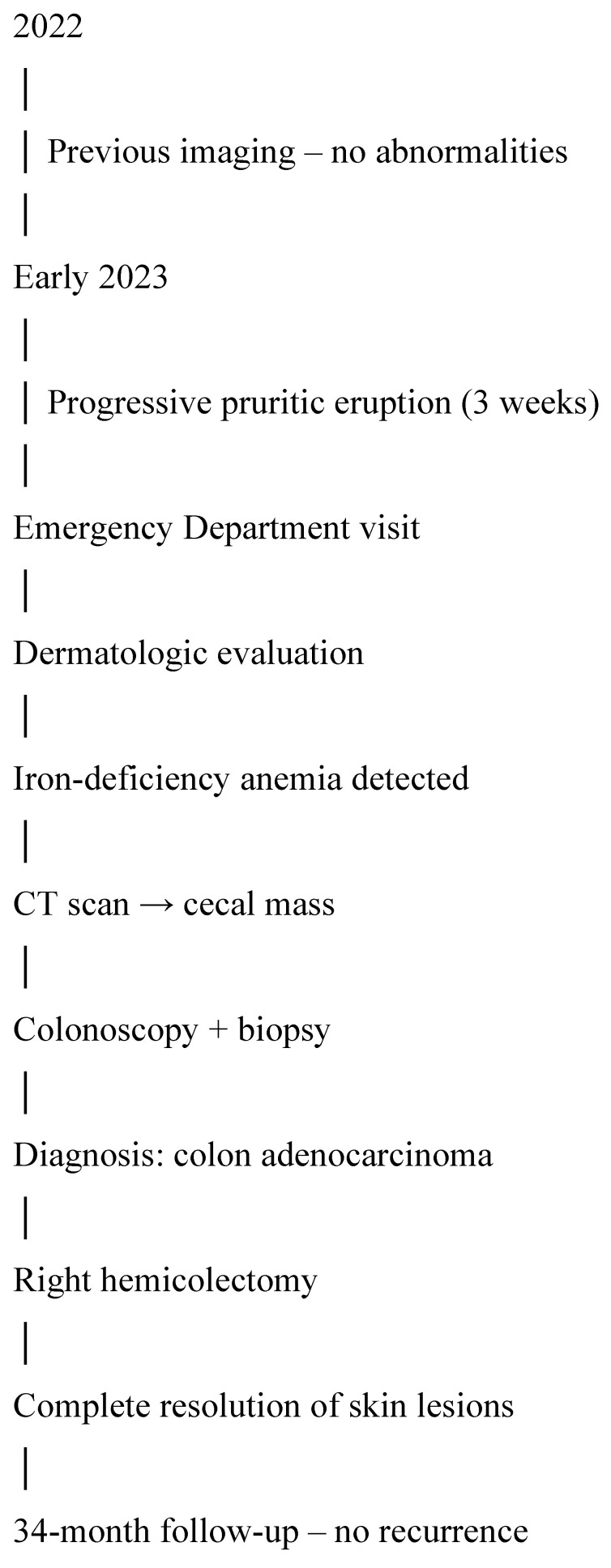
Timeline of clinical events.

**Figure 6:**
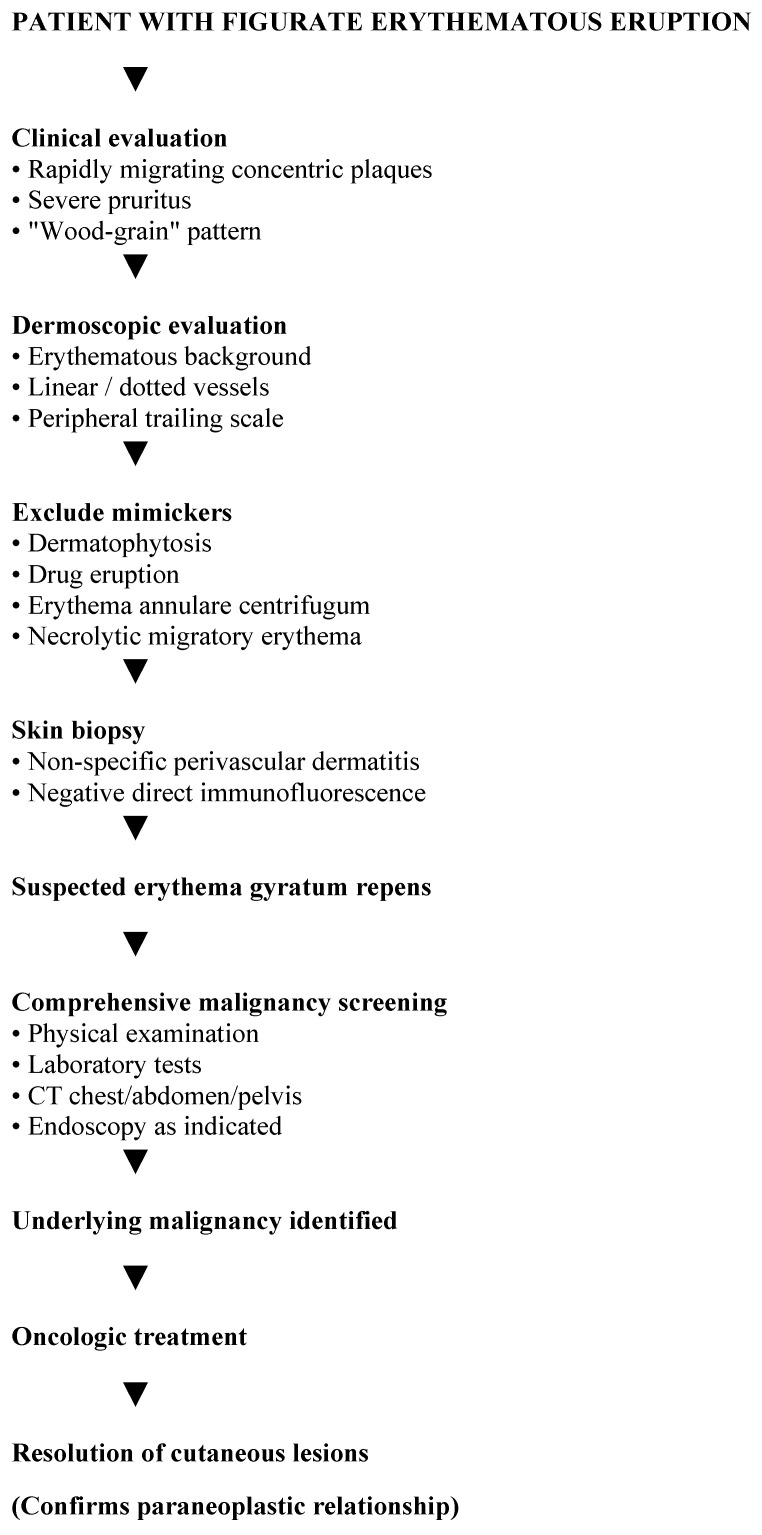
Proposed diagnostic algorithm for suspected erythema gyratum repens.

After surgical resection, the cutaneous lesions completely resolved, supporting the diagnosis of paraneoplastic erythema gyratum repens. At 34 months of follow-up—conducted with regular three-month consultations, CEA levels every three months, CT scans every six months, and a colonoscopy at 12 months post-surgery—the patient remained in good overall health and showed no signs of oncological recurrence or dermatological relapse, with no recurrence of pruritus or figurate erythema. The timeline of clinical events is represented in Figure 5. In Figure 6 we proposed a diagnostic algorithm for suspected erythema gyratum repens.

## 3. Discussion

Erythema gyratum repens (EGR) is a distinctive figurate erythema and remains one of the most specific cutaneous markers of internal malignancy [6]. First described by Gammel in 1952 in association with breast adenocarcinoma, with resolution following tumor excision, EGR has since been recognized as a prototypical paraneoplastic dermatosis [9]. It predominantly affects older adults, with a reported male predominance and a mean age at onset of approximately 60–65 years [7,13]. Most reported cases occur in Caucasian patients, although this may reflect reporting bias rather than true epidemiological differences [13].

Clinically, the eruption is characterized by rapidly migrating, undulating erythematous bands with a distinctive “wood-grain” pattern and a trailing scale, often accompanied by intense pruritus [7,14]. Lesions may advance at a rate of up to 1 cm per day and typically involve the trunk and proximal extremities, with relative sparing of the face, hands, and feet [4,7]. These striking morphological features, as observed in our patient, are central to the diagnosis and frequently allow clinical recognition even in the absence of specific histopathological findings.

Histopathological examination in EGR is characteristically non-specific. Reported findings include mild hyperkeratosis or parakeratosis, focal spongiosis, acanthosis, and a superficial perivascular lymphocytic infiltrate [4,7]. Direct immunofluorescence is usually negative, and no pathognomonic immunohistochemical features have been consistently identified [15,16]. In the present case, a skin biopsy demonstrated a non-specific perivascular dermatitis pattern with negative direct immunofluorescence, consistent with prior reports and reinforcing that the diagnosis of EGR remains primarily clinical.

Erythema gyratum repens is most commonly associated with an underlying malignancy, with more than 80% of reported cases linked to neoplastic disease [6]. Lung cancer represents the most frequent association, followed by esophageal and breast carcinomas; however, a wide range of other malignancies have been described, including gastrointestinal, genitourinary, and hematological cancers [4,7,10,12,13,17]. Although colorectal cancer is less frequently reported, several cases document EGR preceding or coinciding with its diagnosis, underscoring the importance of gastrointestinal evaluation when EGR is identified [10,11,12].

The temporal relationship between EGR and malignancy is clinically relevant. In many cases, the cutaneous eruption precedes the diagnosis of cancer by several months, serving as an early clinical warning sign [4,7]. Less commonly, EGR may occur concurrently with or after cancer diagnosis. Importantly, resolution of the eruption following successful oncologic treatment, as observed in our patient, strongly supports a paraneoplastic etiology, whereas persistence or recurrence of the rash may herald residual disease or tumor relapse [6,8,11].

Although EGR is classically regarded as an obligate paraneoplastic dermatosis, rare non-neoplastic associations have been reported, including infections, autoimmune diseases, drug exposure, pregnancy, and vaccination [7,14,17,18,19,20,21,22,23]. These observations suggest that EGR may represent a broader manifestation of systemic immune dysregulation rather than an exclusively tumor-driven phenomenon. Nevertheless, such associations remain uncommon, and malignancy must always be considered the primary diagnosis until rigorously excluded. Accordingly, EGR should prompt an exhaustive search for occult malignancy, with non-neoplastic causes considered diagnoses of exclusion.

The exact pathogenesis of EGR remains incompletely understood. Several immunopathogenic mechanisms have been proposed to explain the development of erythema gyratum repens. One hypothesis posits that molecular mimicry between tumor-associated antigens and cutaneous structural proteins leads to cross-reactive immune responses. Circulating immune complexes and complement activation have also been described in some cases, leading to inflammatory changes in the dermal–epidermal junction [5,7]. Early immunopathological studies demonstrated occasional deposition of immunoglobulins and complement components at the dermal–epidermal junction, although these findings are inconsistent and lack diagnostic specificity [15,16]. In addition, tumor-derived cytokines, including interleukin-6 and tumor necrosis factor-α, may contribute to keratinocyte activation and the characteristic migratory erythema. These mechanisms support the concept that EGR represents an immune-mediated systemic reaction to malignancy rather than a direct tumor-related skin infiltration. More recent reports highlighting EGR in the context of infections or vaccination further support the hypothesis that disruption of immune homeostasis—whether triggered by malignancy or other systemic insults—may precipitate the characteristic eruption [14,20,21,22].

In the present case, the underlying neoplasm was a well-differentiated, microsatellite-stable adenocarcinoma of the cecum. This finding is noteworthy, as it suggests that a hypermutated or microsatellite instability–high phenotype is not required for the development of EGR. Instead, alternative mechanisms—such as humoral immune responses, circulating tumor-associated antigens, or cytokine-driven effects—may be sufficient to trigger the cutaneous manifestation [16,24]. This observation reinforces the notion that the absence of high-risk molecular features does not preclude a paraneoplastic presentation. This further supports the concept that EGR is not restricted to biologically aggressive or hypermutated tumors.

From a clinical perspective, this case underscores the importance of dermatologic pattern recognition in the emergency setting. The identification of a characteristic figurate erythema, combined with systemic red flags such as iron-deficiency anemia and unintentional weight loss, appropriately directs diagnostic efforts toward an underlying malignancy. In this context, extensive immunological investigations were not pursued, as the clinical suspicion of neoplasia was high and subsequently confirmed by imaging and endoscopic evaluation.

Finally, this case supports EGR’s role as a dynamic marker of disease activity. The complete resolution of cutaneous lesions following surgical resection and the absence of dermatologic or oncological recurrence during long-term follow-up further highlight its potential utility not only as a diagnostic clue but also as an indicator of treatment response and disease control.

## 4. Conclusions

Erythema gyratum repens is a rare but highly specific paraneoplastic dermatosis whose recognition may directly lead to the early diagnosis of internal malignancy. Owing to its characteristic morphology and rapid progression, it represents a clinically actionable cutaneous signal that may precede or coincide with the detection of an otherwise occult neoplasm.

This case illustrates erythema gyratum repens as the sentinel manifestation of a right-sided, microsatellite-stable colon adenocarcinoma diagnosed in the emergency setting. The complete resolution of the cutaneous eruption following curative surgical resection, together with the absence of dermatologic or oncologic recurrence during long-term follow-up, supports a true paraneoplastic relationship and highlights this dermatosis as a dynamic marker of tumor activity rather than a static dermatologic finding.

From a clinical standpoint, prompt recognition of distinctive figurate erythema—particularly when accompanied by systemic red flags such as iron-deficiency anemia or unintentional weight loss—should prompt a comprehensive malignancy workup. Increased awareness of erythema gyratum repens among dermatologists, surgeons, internists, and emergency physicians may therefore facilitate earlier cancer detection, guide timely definitive treatment, and improve patient outcomes.

### Future Research Directions

Future research should prioritize developing screening strategies for patients presenting with figurate erythema, particularly in emergency departments and outpatient dermatology settings, where early diagnostic decisions are often made. Prospective, multicenter studies integrating structured dermatologic assessment with targeted imaging and endoscopic evaluation may help define the most efficient and cost-effective diagnostic pathways and establish clear thresholds for gastrointestinal investigation in the absence of overt digestive symptoms.

In parallel, further investigation into the immunological mechanisms underlying erythema gyratum repens is warranted. Future studies should aim to clarify the relative contributions of humoral immune responses, cytokine-driven inflammation, and tumor antigen cross-reactivity in disease initiation and resolution. Standardized immunological profiling—including cytokine panels, immunohistochemical markers, and direct immunofluorescence—may help identify reproducible immune signatures associated with disease activity and remission, thereby improving mechanistic understanding and diagnostic confidence.

Finally, translational research integrating dermatologic findings with immunologic and oncologic parameters could enhance the role of erythema gyratum repens as a dynamic biomarker for disease monitoring, recurrence detection, and treatment response assessment. While targeted immunomodulatory therapies, such as biologic agents or Janus kinase inhibitors, may merit exploration in carefully selected non-paraneoplastic or refractory cases, their use should be guided by robust immunological characterization and a clear understanding of underlying disease drivers.

## Figures and Tables

**Table 1:** Clinical features of selected dermatoses aiding in the differential diagnosis of erythema gyratum repens.

Condition	Key Clinical Features	Distinguishing Clues
Erythema annulare centrifugum	Slowly expanding annular plaques	Trailing scale but slower migration
Necrolytic migratory erythema	Erosive annular plaques	Associated with glucagonoma
Drug eruption	Variable morphology	Temporal relation to drug exposure
Dermatophytosis	Annular lesions with scaling	Positive fungal microscopy
Eczema	Pruritic erythematous plaques	No concentric migration

## Data Availability

The original contributions presented in this study are included in the article. Further inquiries can be directed to the corresponding author.

## Supplementary Materials

The following supporting information can be downloaded at: https://www.mdpi.com/article/10.3390/jcm15072789/s1, Figure S1: Representative image of the skin biopsy showing features consistent with perivascular dermatitis (H&E, 100x).
