# Supplementary material for: Cutaneous Manifestations as a Sentinel of Colorectal Cancer: A Case Report
Source: J Clin Med. 2026 Apr 7;15(7):2789. doi: 10.3390/jcm15072789 (PMC13073435; doi:10.3390/jcm15072789)
Supplement: Supplementary file 1 [file jcm-15-02789-s001.zip › jcm-4159007-supplementary.pdf]

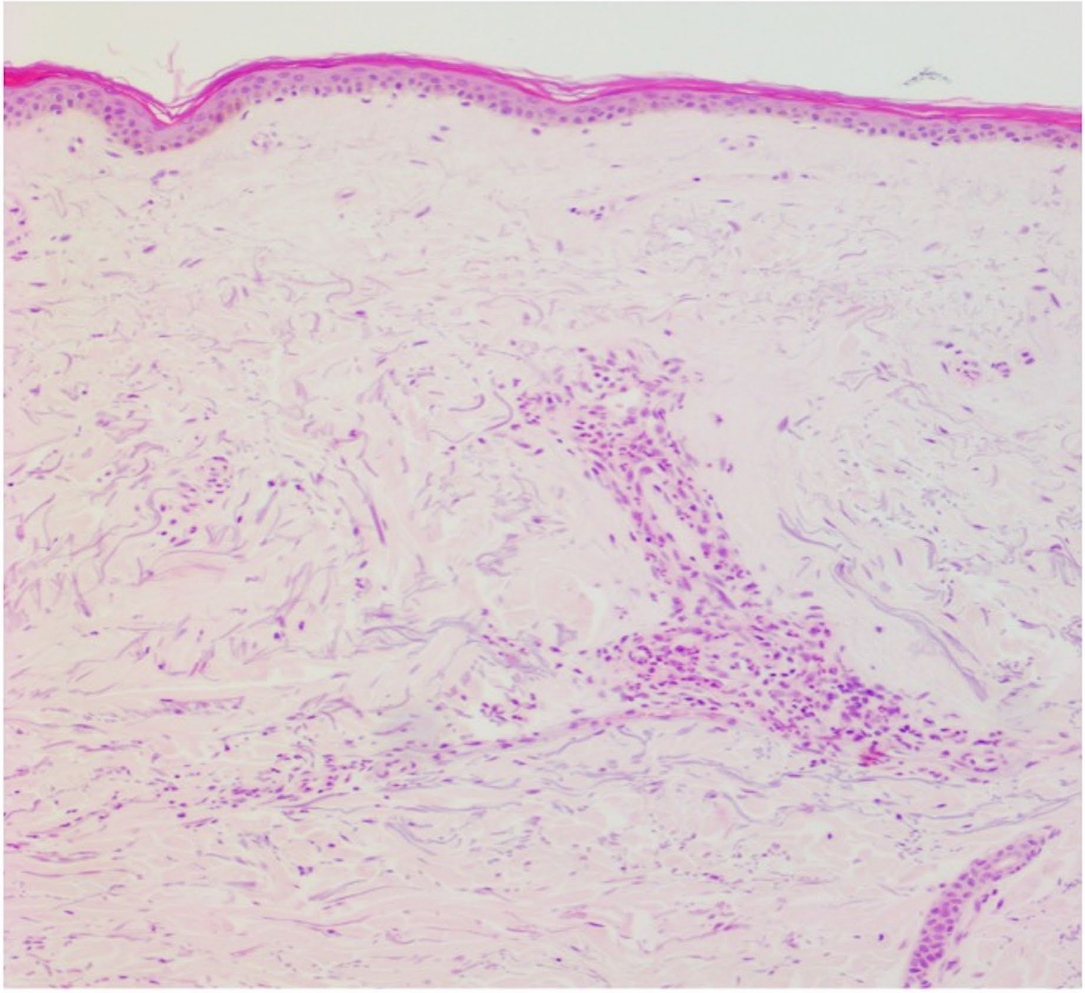

Figure S1. Representative image of the skin biopsy showing features consistent with perivascular dermatitis (H&E, 100x).
